# Soziodemografische und soziale Korrelate selbstberichteter Resilienz im Alter – Ergebnisse der populationsbasierten LIFE-Adult-Studie

**DOI:** 10.1007/s00103-023-03675-7

**Published:** 2023-03-06

**Authors:** Elena Caroline Weitzel, Heide Glaesmer, Andreas Hinz, Samira Zeynalova, Sylvia Henger, Christoph Engel, Markus Löffler, Nigar Reyes, Kerstin Wirkner, A. Veronica Witte, Arno Villringer, Steffi G. Riedel-Heller, Margrit Löbner

**Affiliations:** 1 Institut für Sozialmedizin, Arbeitsmedizin und Public Health (ISAP), Medizinische Fakultät, Universität Leipzig, Philipp-Rosenthal-Str. 55, 04103 Leipzig, Deutschland; 2 Abteilung für Medizinische Psychologie und Medizinische Soziologie, Medizinische Fakultät, Universität Leipzig, Leipzig, Deutschland; 3 Institut für Medizinische Informatik, Statistik und Epidemiologie, Universität Leipzig, Leipzig, Deutschland; 4 LIFE – Leipziger Forschungszentrum für Zivilisationserkrankungen, Universität Leipzig, Leipzig, Deutschland; 5 Abteilung Neurologie, Max-Planck-Institut für Kognitions- und Neurowissenschaften, Max-Planck-Gesellschaft, Leipzig, Deutschland

**Keywords:** Prävention, Alter, Ressourcen, Soziales Netz, Soziale Unterstützung, Prevention, Old age, Resources, Social network, Social support

## Abstract

**Einleitung:**

Resilienz bezeichnet eine gute Anpassung an Widrigkeiten und ist ein bedeutsamer Faktor für das Wohlbefinden im Alter. Erste Studien weisen auf eine hohe Relevanz sozialer Ressourcen hin. Bisher haben nur wenige Studien Resilienzmuster in der Altenbevölkerung untersucht. Die vorliegende Studie hat zum Ziel, soziodemografische und soziale Korrelate von Resilienz in einer großen populationsbasierten Stichprobe ab 65 Jahren zu identifizieren.

**Methoden:**

Analysiert wurden Daten von *n* = 2410 Menschen ab 65 Jahren aus der LIFE-Adult Studie. Erhoben wurden Daten zu Resilienz (Resilienzskala – RS-11), sozialer Unterstützung (ENRICHD Social Support Inventory – ESSI) und zum sozialen Netz (Lubben Social Network Scale – LSNS-6). Der Zusammenhang soziodemografischer und sozialer Variablen mit Resilienz wurde mittels einer multiplen linearen Regressionsanalyse analysiert.

**Ergebnisse:**

Das Alter ab 75 Jahren war mit einer niedrigeren Resilienz im Vergleich zum Alter von 65 bis 74 Jahren assoziiert. Der Familienstand „verwitwet“ hing mit einer höheren Resilienz zusammen. Eine bessere soziale Unterstützung und ein größeres soziales Netz waren mit einer höheren Resilienz assoziiert. Kein Zusammenhang wurde für die Variablen Geschlecht und Bildung gefunden.

**Diskussion:**

Die Ergebnisse zeigen soziodemografische Korrelate von Resilienz in der Altenbevölkerung auf, die zur Identifizierung von Risikogruppen mit niedrigerer Resilienz beitragen können. Soziale Ressourcen sind im höheren Alter für eine resiliente Anpassung bedeutsam und stellen einen Ansatzpunkt zur Ableitung von Präventionsmaßnahmen dar. Die soziale Einbindung älterer Menschen sollte gefördert werden, um Resilienz in dieser Bevölkerungsgruppe zu stärken und günstige Bedingungen für ein erfolgreiches Altern zu schaffen.

## Abstract (en)

**Introduction:**

Resilience describes good adaptation to adversity and is a significant factor for well-being in old age. Initial studies indicate a high relevance of social resources. So far, only few studies have investigated resilience patterns in the elderly population. Therefore, the present study aims to investigate sociodemographic and social correlates of resilience in a large population-based sample aged 65 years and older.

**Methods:**

Analyses were conducted on *n* = 2410 people aged 65 years and older from the follow-up survey of the LIFE-Adult-Study. The survey included the variables resilience (Resilience Scale – RS-11), social support (ENRICHD Social Support Inventory – ESSI), and social network (Lubben Social Network Scale – LSNS-6). The association of sociodemographic and social variables with resilience was analyzed using multiple linear regression analysis.

**Results:**

The age of 75 years and older was associated with lower resilience compared with the age of 65–74 years. Further, widowed marital status was related to higher resilience. Better social support and a larger social network were significantly associated with higher resilience. No association was found for gender and education.

**Discussion:**

The results reveal sociodemographic correlates of resilience in the elderly population that can help identify at-risk groups with lower resilience. Social resources are significant in older age for resilient adaptation and represent a starting point for deriving preventive measures. Social inclusion of older people should be promoted to strengthen resilience in this population and provide favorable conditions for successful aging.

## Einleitung

Resilienz bedeutet eine Bewahrung und/oder Wiederherstellung des psychosozialen Funktionsniveaus angesichts schwieriger oder herausfordernder Lebenskontexte, z. B. durch Stress, Bedrohungserleben oder Verluste [1]. Das Konstrukt kennzeichnet eine Hinwendung zu Ressourcen und protektiven Faktoren im Kontext psychischer Gesundheit [2, 3]. Wie sich Resilienz konzeptualisiert, wurde umfassend diskutiert [4]. Dabei wurde Resilienz teilweise als Persönlichkeitsmerkmal begriffen, welches Individuen innewohnt und sie kritische Lebensereignisse besser überstehen lässt [5]. Inzwischen dominiert die Auffassung von Resilienz als Prozess, der in Interaktion von persönlichen Eigenschaften und Fähigkeiten, externen Ressourcen und dem aversiven Ereignis stattfindet [4, 6–8]. Bestimmte persönliche Eigenschaften, wie etwa Optimismus und Sinnhaftigkeit, sind Resilienz dennoch dienlich [9]. Als eher externe Ressourcen spielen unter anderem der sozioökonomische Status und die soziale Unterstützung eine bedeutsame Rolle [9, 10].

Das komplexe Zusammenspiel externer und interner Ressourcen und Stressoren birgt methodische Herausforderungen in der Operationalisierung von Resilienz. In einigen Studien wurde Resilienz als Abwesenheit von Symptomen erfasst [11, 12], jedoch bedeutet (kurzzeitige) Beeinträchtigung nicht zwangsläufig eine geringe Resilienz. Andere erheben Resilienz über positive Korrelate wie die Lebenszufriedenheit [13], was die inhaltliche Abgrenzung der Konzepte erschwert [2]. Um der individuellen Konstellation von Ressourcen und Stressoren und der Komplexität des Konstrukts gerecht zu werden, wird Resilienz in dieser Studie im Sinne eines subjektiven Resilienzerlebens begriffen und als explizites Konstrukt im Selbstbericht erhoben.

Eine besondere Bedeutung kommt Resilienz in der Altenbevölkerung ab 65 Jahren zu. Hier steigt die Auftretenswahrscheinlichkeit von Krankheiten, zwischenmenschlichen Verlusten und Funktionseinschränkungen, die Anpassungsleistungen erfordern [14]. Ein steigendes Alter geht jedoch nicht mit einem schlechteren Befinden einher, vielmehr berichten ältere Menschen im Allgemeinen eine hohe Lebenszufriedenheit [15], ein stabiles Wohlbefinden [16] und erleben ihr Altern trotz körperlicher und kognitiver Beeinträchtigungen subjektiv als erfolgreich [17]. Dass im höheren Alter eine hohe Resilienz vorliegt, wird durch bisherige Studien empirisch untermauert [5, 18]. So war die Altenbevölkerung zuletzt auch während der COVID-19-Pandemie trotz höheren Gesundheitsrisikos durch das Coronavirus weniger psychisch belastet als jüngere Bevölkerungsgruppen [19].

Ein besseres Verständnis von Resilienzmustern in der Altenbevölkerung kann Ansatzpunkte für Präventionsangebote liefern, die sich speziell an vulnerable Gruppen richten. Bisherige Studien in der deutschen Altenbevölkerung weisen auf ein beeinträchtigtes Wohlbefinden bei Frauen und bei Hochaltrigen hin [20]. Die bisher wenigen Studien zu Korrelaten selbstberichteter Resilienz beziehen sich häufig auf besonders exponierte Subgruppen und liefern heterogene Befunde zum Zusammenhang des Resilienzerlebens mit dem Alter, dem Geschlecht und der Bildung [10, 21–24]. Großangelegte populationsbasierte Studien in der deutschen Altenbevölkerung stehen aus.

Weiter ist die Einbindung sozialer Ressourcen in Studien zur Resilienz im Alter notwendig. Die Verfügbarkeit sozialer Ressourcen verändert sich mit dem Alter und stellt einen relevanten Faktor für die Lebenszufriedenheit und psychische Gesundheit älterer Menschen dar [25, 26]. Soziale Ressourcen hängen maßgeblich mit der selbstberichteten Resilienz zusammen [27]. Erste Studien in anderen Ländern zeigen eine hohe Bedeutsamkeit von sozialen Ressourcen für selbstberichtete Resilienz in älteren Stichproben [28, 29]. Inwieweit die Befunde dieser Studien auf die deutsche Altenbevölkerung übertragbar sind, ist unklar, da die Bedeutung sozialer Kontakte auch von der jeweiligen Kultur abhängt [30]. Die Relevanz sozialer Ressourcen für Resilienz ist in der deutschen Altenbevölkerung ab 65 Jahren bisher nicht untersucht worden, könnte aber wertvolle Informationen für die Ableitung wirksamer Unterstützungsangebote liefern.

Um Resilienzmuster im Alter aufzudecken und altersspezifische Resilienzfaktoren besser zu verstehen, besteht Forschungsbedarf zu Korrelaten selbstberichteter Resilienz in der Altenbevölkerung, die soziale Ressourcen miteinbeziehen. Mithilfe eines besseren Verständnisses von Resilienzmustern in der Altenbevölkerung könnten vulnerable Gruppen identifiziert und Resilienz zielgerichtet gefördert werden. Daher sollen in dieser Studie soziodemografische und soziale Korrelate von selbstberichteter Resilienz in der deutschen Altenbevölkerung ab 65 Jahren untersucht werden.

## Methoden

### Stichprobe

Die Daten für die Untersuchung stammen aus der populationsbasierten LIFE Adult-Studie des Leipziger Forschungszentrum für Zivilisationserkrankungen (LIFE), einer großangelegten Kohortenstudie mit 10.000 Teilnehmenden in Leipzig [31, 32]. Die vorliegende querschnittliche Analyse wurde mit Daten der ersten Nachbefragung durchgeführt, in welcher Resilienz erstmalig erhoben wurde. Die Nachbefragung fand 2017 bis 2021 in Leipzig statt.

Für die Analyse waren Nacherhebungsdaten von *n* = 3011 Teilnehmenden im Alter von 65 Jahren und älter verfügbar. Von diesen wurden 366 wegen fehlender Angaben in den soziodemografischen und sozialen Variablen ausgeschlossen. Insgesamt *n* = 75 wurden wegen fehlender Angaben im Resilienzfragebogen ausgeschlossen. Die Analysestichprobe dieser Studie belief sich damit auf *n* = 2410.

### Datenerhebung

*Soziodemografische Informationen* lagen zu Alter, Geschlecht, Familienstand und zur bildungs- und berufsbezogenen Qualifikation vor. Für eine bessere Aussagekraft der Ergebnisse fassten wir die Studienteilnehmenden in Anlehnung an Gao et al. (2019; [33]) ursprünglich in den Altersgruppen 65–74 Jahre, 75–84 Jahre und ≥ 85 Jahre zusammen. Aufgrund der sehr niedrigen Anzahl von Studienteilnehmenden mit einem Alter von 85 Jahren oder älter (*n* = 34) mussten die beiden älteren Gruppen jedoch zur Altersgruppe ≥ 75 Jahre und älter subsumiert werden. Das Bildungsniveau von niedrig, mittel und hoch wurde mithilfe der Bildungsklassifikation „Comparative Analysis of Social Mobility in Industrial Nations“ (CASMIN) bestimmt [34].

*Resilienz* wurde mithilfe der deutschen Kurzversion der Resilienzskala (RS-11) von Wagnild und Young erhoben [2, 35]. Die RS-11 misst Resilienz als unidimensionales Konstrukt und zeichnet sich durch eine hohe Validität und sehr gute psychometrische Kennwerte aus (11 Items, Cronbachs Alpha = 0,91; [2]). Sie ist laut Cosco et al. (2016) für die Untersuchung von Resilienz in älteren Stichproben geeignet [36]. In unserer Stichprobe ab 65 Jahren betrug die Reliabilität der 11 Items insgesamt 0,91 (Cronbachs Alpha) und für die Altersgruppen jeweils 0,90 (65–74 Jahre) und 0,91 (ab 75 Jahren). Die 11 Items können auf einer Skala von 1 = „Ich stimme nicht zu“ bis 7 = „Ich stimme völlig zu“ beantwortet werden. Items sind bspw.: „Normalerweise schaffe ich alles irgendwie“, „Normalerweise kann ich eine Situation aus mehreren Perspektiven betrachten“ und „Ich kann mehrere Dinge gleichzeitig bewältigen“. Alle Antworten werden zu einem Summenscore mit der Spannweite 11–77 aufaddiert, bei dem höhere Werte eine höhere Resilienz bedeuten [2].

Die *soziale Unterstützung* wurde mit dem ENRICHD Social Support Inventory (ESSI) gemessen [37, 38]. Die 5 Items erfragen die Erreichbarkeit von unterstützenden Kontakten, also von Menschen, denen man Vertrauen entgegenbringt und die Zuneigung und Liebe spenden. Aus den Antworten von 1 = „nie“ bis 5 = „immer“ wird ein Summenscore gebildet, der höher ausfällt, je mehr soziale Unterstützung vorliegt.

Um die *Größe des sozialen Netzes* abzubilden wurde die Kurzversion der Lubben Social Network Scale (LSNS-6) eingesetzt. Mithilfe von 6 Items werden quantitative Informationen zur Anzahl und zum Kontakt mit familiären und freundschaftlich verbundenen Bezugspersonen erfasst, die für Hilfe und private Gespräche zur Verfügung stehen. Die Antworten werden auf einer Skala mit den Optionen 0 = „keine“, 1 = „1“, 2 = „2“, 3 = „3 oder 4“, 4 = „5 bis 8“ und 5 = „9 oder mehr“ gegeben. Es wird ein Summenscore berechnet, wobei höhere Werte ein größeres soziales Netz anzeigen.

### Statistische Analysen

Der Zusammenhang von Resilienz und soziodemografischen und sozialen Korrelaten wurde mittels einer multiplen linearen Regressionsanalyse geprüft. Resilienz wurde als abhängige Variable definiert und Geschlecht, Altersgruppe, Familienstand, Bildung, soziale Unterstützung und soziales Netz als unabhängige Variablen. Dabei zählten die folgenden Ausprägungen als Referenzkategorie: männliches Geschlecht, Altersgruppe 65–74 Jahre, Familienstand „verheiratet“ und niedriges Bildungsniveau. Die kontinuierlichen Variablen wurden für die Regressionsanalyse mittelwertszentriert und über die Standardabweichung (SD) für eine bessere Vergleichbarkeit skaliert. Die Prädiktorvariablen wurden anhand der Varianzinflationsfaktoren (VIF) auf Multikollinearität geprüft.

Alle Analysen wurden mit der Software R [39] bzw. RStudio [40] durchgeführt. Die statistische Signifikanz wurde mit *p* < 0,05 definiert.

## Ergebnisse

### Stichprobencharakteristika.

Wie in Tab. 1 aufgeführt waren die Teilnehmenden im Mittel 73,9 Jahre (*SD* = 5,5) alt und 50,5 % (*n* = 1217) waren weiblich. Das Durchschnittsalter der Frauen betrug *M* = 73,6 Jahre (*SD* = 5,4), die Männer waren durchschnittlich 74,2 Jahre (*SD* = 5,5) alt. Die Altersgruppe der 65- bis 74-Jährigen war geringfügig größer als die der ab 75-Jährigen (52,6 %, *n* = 1267 vs. 47,4 %, *n* = 1143). Mit 70,6 % (*n* = 1701) wies der Großteil der Teilnehmenden den Familienstand „verheiratet“ auf, am kleinsten war die Gruppe der ledigen Teilnehmenden (3,6 %, *n* = 87). Die Teilnehmenden waren überwiegend mittelgradig (47,8 %, *n* = 1151) und hoch gebildet (42,3 %, *n* = 1020).

**Table 1:** 

	Gesamt *n* = 2410		Frauen *n* = 1217		Männer *n* = 1193	
Alter, *M* (*SD*)	73,9	(5,5)	73,6	(5,4)	74,2	(5,5)
*Altersgruppe, n (%)*						
65–74 Jahre	1267	(52,6)	662	(54,4)	605	(50,7)
≥ 75 Jahre	1143	(47,4)	555	(45,6)	588	(49,3)
*Familienstand, n (%)*						
Verheiratet	1701	(70,6)	714	(58,7)	987	(82,7)
Ledig	87	(3,6)	49	(4,0)	38	(3,2)
Geschieden	246	(10,2)	163	(13,4)	83	(7,0)
Verwitwet	376	(15,6)	291	(23,9)	85	(7,1)
*Bildungsniveau, n (%)*						
Niedrig	239	(9,9)	152	(12,5)	87	(7,3)
Mittel	1151	(47,8)	688	(56,5)	463	(38,8)
Hoch	1020	(42,3)	377	(31,0)	643	(53,9)

*M* Mittelwert, *SD* Standardabweichung

### Resilienz.

Der Resilienzmittelwert auf der RS-11 betrug in der gesamten Stichprobe 60,3 (*SD* = 11,5). In Tab. 2 sind die Mittelwerte der Resilienz in den Altersgruppen und nach Geschlecht aufgelistet. Resilienz war in der Altersgruppe der 65- bis 74-Jährigen mit einem Mittelwert von 61,4 (SD = 11,0) höher als in der Altersgruppe ab 75 Jahren (*M* = 59,1; *SD* = 11,8) ausgeprägt (*t*(2340,3) = 4,907, *p* < 0,001). Die mittlere Resilienz unterschied sich nicht signifikant zwischen Männern und Frauen (*t*(2408) = −1,44, *p* = 0,151). Am höchsten war die Resilienz in der Gruppe der 65- bis 74-jährigen Frauen ausgeprägt (*M* = 61,5; *SD* = 11,5), am niedrigsten in der Gruppe der Männer im Alter von 75 Jahren und älter (*M* = 58,6; *SD* = 12,0).

**Table 2:** 

		Gesamt *n* = 2410		Frauen *n* = 1217		Männer *n* = 1193	
	*n*	*M*	*SD*	*M*	*SD*	*M*	*SD*
Gesamt	–	60,3	11,5	60,7	11,6	60,0	11,3
*Altersgruppe*							
65–74 Jahre	1267	61,4	11,0	61,5	11,5	61,3	10,5
≥75 Jahre	1143	59,1	11,8	59,6	11,6	58,6	12,0

Resilienz wurde mit der RS-11 erfasst (Spannweite: 11–77)

*M* Mittelwert, *SD* Standardabweichung

### Soziodemografische und soziale Resilienzkorrelate.

Tab. 3 stellt die Interkorrelationen der metrischen Studienvariablen dar. Bis auf den Zusammenhang von Alter und sozialer Unterstützung waren alle Interkorrelationen signifikant. Die Ergebnisse der multiplen linearen Regressionsanalyse werden in Tab. 4 dargestellt. Die Modellgüte ermittelt über das Pseudo‑R^2^ nach Cragg-Uhler lag bei 0,133. Signifikante Prädiktoren von Resilienz waren die Altersgruppe, der Familienstand und die soziale Unterstützung sowie das soziale Netz. Dabei war die Altersgruppe ab 75 Jahren im Vergleich zur Altersgruppe von 65–74 Jahren mit einer niedrigeren Resilienz assoziiert (β = −0,169, *p* < 0,001). Verwitwung war in Referenz zum Familienstand „verheiratet“ mit einer höheren Resilienz assoziiert (β = 0,161, *p* = 0,004). Weiter sagten eine höhere soziale Unterstützung und ein größeres soziales Netz jeweils eine höhere Resilienz vorher (β = 0,265, *p* < 0,001 und β = 0,139, *p* < 0,001). Kein signifikanter Unterschied im Vergleich zur Referenzgruppe wurde für das weibliche Geschlecht (β = 0,033, *p* = 0,418) und ein mittleres (β = 0,080, *p* = 0,251) bzw. höheres Bildungsniveau (β = 0,129, *p* = 0,068) gefunden.

**Table 3:** 

	M	SD	Alter	Resilienz	Soziale Unterstützung
Alter	73,9	5,5	1	–	–
Resilienz	60,3	11,5	−0,10**	1	–
Soziale Unterstützung	22,3	3,5	0,01	0,30**	1
Soziales Netz	16,1	5,4	−0,12**	0,24**	0,36**

Resilienz, soziale Unterstützung und das soziale Netz wurden mit der RS-11, ESSI und LSNS‑6 erfasst

**p < 0,001

**Table 4:** 

	β	95 %-KI Untergrenze	95 %-KI Obergrenze	*p*	VIF
**Soziodemografische Prädiktoren**					
*Altersgruppe (Referenzgruppe: 65–74 Jahre)*					
≥75 Jahre	−0,169	−0,248	−0,090	<0,001	1,114
Geschlecht (Referenzgruppe: männlich)					
Weiblich	0,033	−0,047	0,114	0,418	1,156
*Familienstand (Referenzgruppe: verheiratet)*					
Ledig	0,158	−0,048	0,365	0,133	1,217
Geschieden	0,125	−0,005	0,254	0,059	1,217
Verwitwet	0,161	0,051	0,272	0,004	1,217
*Bildungsniveau (Referenzgruppe: niedrig)*					
Mittel	0,080	−0,056	0,215	0,251	1,170
Hoch	0,129	−0,009	0,267	0,068	1,170
**Soziale Prädiktoren**					
Soziale Unterstützung	0,265	0,224	0,306	<0,001	1,207
Soziales Netz	0,139	0,098	0,180	<0,001	1,214

Resilienz, soziale Unterstützung und soziales Netz wurden mit der RS-11, dem ENRICHD Social Support Inventory (ESSI) und der Lubben Social Network Scale (LSNS-6) erfasst. Regressionsgewichte β sind standardisiert

*KI* Konfidenzintervall, *VIF* Varianzinflationsfaktor

^a^Die multiple lineare Regressionsanalyse wurde mit Resilienz als abhängiger Variable und soziodemografischen und sozialen Variablen als Prädiktorvariablen durchgeführt

## Diskussion

In der vorliegenden Studie wurden soziodemografische und soziale Korrelate von selbstberichteter Resilienz in einer umfassenden populationsbasierten Stichprobe ab 65 Jahren untersucht. In der altersspezifischen Substichprobe der LIFE-Adult-Studie lag ein Durchschnittswert von 60,3 (*SD* = 11,5) vor, der vergleichbar zu den Durchschnittswerten der Allgemeinbevölkerung bzw. geringfügig höher ausfiel (59,6 (*SD* = 10,65; [41]), 58,03 (*SD* = 10,8; [2])). Damit sprechen die Befunde für ein im Alter fortbestehendes Resilienzerleben gegenüber Belastungen.

In unserer Studie war Resilienz in der Altersgruppe ab 75 Jahren niedriger ausgeprägt als in der Gruppe der 65- bis 74-Jährigen. Diese deskriptiven Ergebnisse wurden in der Regressionsanalyse bestätigt, in der unter Kontrolle von anderen soziodemografischen und sozialen Variablen das Alter ab 75 Jahren mit einer niedrigeren Resilienz assoziiert war. Die bisherige Studienlage zum Zusammenhang von Resilienz mit dem Alter innerhalb älterer Stichproben war heterogen [10, 22, 24]. Einige Studien fanden keine Altersunterschiede oder eine höhere Resilienz in älteren Altersgruppen [17, 22]. Unsere Ergebnisse stehen in Einklang mit Pechmann et al. (2014) und Kocalevent et al. (2015), die eine niedrigere Resilienz in der Altersgruppe der Menschen ab 75 Jahren im Vergleich zu jüngeren Altersgruppen beobachteten [10, 41]. Auch Wohlbefinden und Lebenszufriedenheit sind in der Altenbevölkerung mit steigendem Alter niedriger ausgeprägt [20].

Ursachen für eine niedrigere Resilienz im Alter ab 75 Jahren können vielfältig sein [14]. Zum einen kommen belastende Lebensereignisse wie chronische Erkrankungen, Pflegebedürftigkeit des Ehepartners oder der Verlust nahestehender Personen im Alter ab 75 Jahren häufiger vor und können das Resilienzerleben beeinträchtigen. Auch altersspezifische Einschränkungen im alltäglichen Leben werden mit höherem Alter zunehmend relevant [42]. Mit Blick auf die heterogene Befundlage und eine hohe interindividuelle Variabilität in Hinblick auf altersbezogene Veränderungen sollten weitere Langzeitstudien den Verlauf von Resilienz im höheren Alter unter Einbezug belastender Lebensereignisse und der Funktionsfähigkeit umfassend beleuchten.

In Hinblick auf das Geschlecht konnten wir in unserer Studie keinen Zusammenhang mit Resilienz finden. Damit stehen unsere Ergebnisse in Einklang mit Nygren et al. (2005) und Perna et al. (2012), die keinen Geschlechtsunterschied in älteren Stichproben finden [21, 23]. Ergebnisse aus Studien, die älteren Frauen eine höhere Resilienz zuschreiben (z. B. [24]) sind möglicherweise auf den Verzicht einer statistischen Kontrolle für soziale Ressourcen zurückzuführen, die bei Frauen im höheren Alter stärker ausgeprägt sein können [9]. In anderen Studien zeigten sich ältere Frauen tendenziell stärker psychisch belastet und wiesen ein niedrigeres Wohlbefinden auf als männliche Altersgenossen [20, 43]. Die Diskrepanz der Befunde verdeutlicht, dass selbstberichtete Resilienz, Wohlbefinden und psychische Gesundheit verschiedene Konstrukte darstellen und eine differenzierte Operationalisierung in künftigen Studien beibehalten werden sollte. Unsere Ergebnisse legen nahe, dass sich ein beeinträchtigtes psychisches Wohlbefinden bei Frauen nicht auf ein unterschiedliches Resilienzerleben zurückführen lässt bzw. sich umgekehrt nicht in einem geringeren Resilienzerleben bei Frauen äußert. Künftige Studien sollten den Zusammenhang von selbstberichteter Resilienz zur psychischen Gesundheit sowie geschlechtsspezifische Mechanismen untersuchen.

In Bezug auf den Familienstand stellten wir eine höhere Resilienz bei verwitweten und entsprechend eine niedrigere Resilienz bei verheirateten Studienteilnehmenden fest. Diese Ergebnisse stehen in Widerspruch zu anderen Studien, die eine geringe Resilienz in unmittelbarer Folge des Verlusts des Ehepartners feststellen [44]. Diese Diskrepanz ist möglicherweise auf altersspezifische Besonderheiten und die querschnittliche Erhebung in unserer Analyse zurückzuführen. So kann einerseits seit dem Verlust des Ehepartners bereits eine längere Zeit verstrichen sein und der Umgang mit der Verlusterfahrung das Resilienzerleben im Sinne eines persönlichen Wachstums gestärkt haben. Andererseits ist bei verheirateten Menschen in höherem Alter die Wahrscheinlichkeit der häuslichen Pflege des Ehepartners deutlich erhöht. So werden in Deutschland über 40 % der 70- bis 85-Jährigen durch den Ehepartner oder die Ehepartnerin unterstützt und gepflegt [45]. Häusliche Pflege und Krankheit des Ehepartners oder der Ehepartnerin stellen eine hohe chronische Belastung dar, die Resilienzressourcen deutlich beeinträchtigen können [46]. Künftige Studien zur Resilienz im Alter sollten die häusliche Pflege miteinbeziehen.

Das Bildungsniveau hing in unserer Studie nicht bedeutsam mit Resilienz zusammen. Damit bestätigen unsere Befunde Studien von Wells (2010) und Netuveli et al. (2008), in denen kein Unterschied in der Resilienz nach Bildung gefunden wurde [24, 29]. In anderen Studien wurde hingegen ein Zusammenhang höherer Bildungsabschlüsse mit hoher Resilienz im Alter gefunden [21, 22]. Da auch in unserer Studie eine Tendenz zu einer höheren Resilienz bei hohem Bildungsniveau beobachtet werden konnte (β = 0,129, *p* = 0,068), sollte der Zusammenhang von Bildung und Resilienz im Alter in künftigen Studien spezifischer untersucht werden. Möglicherweise könnte eine bessere soziale Inklusion bei höherem Bildungsniveau einen Zusammenhang vermitteln [47], wofür in unserer Auswertung statistisch kontrolliert wurde.

Einen deutlichen Zusammenhang konnten wir zwischen sozialer Unterstützung und der Größe des sozialen Netzes mit Resilienz feststellen. Damit bestätigen unsere Ergebnisse bisherige Hinweise auf eine Bedeutsamkeit zwischenmenschlicher Beziehungen für Resilienz im Alter [28, 29]. Besonders die soziale Unterstützung hing in unserer Studie eng mit dem Resilienzerleben von Menschen ab 65 Jahren zusammen. Auch die Größe des sozialen Netzes ist für Resilienz relevant. Gerade im Alter ist durch den Renteneintritt, chronische Erkrankungen und den Verlust nahestehender Personen [14] eine Verringerung sozialer Kontakte möglich und Einsamkeit häufig [48]. Unsere Ergebnisse verdeutlichen, dass die Förderung der sozialen Einbindung älterer Menschen einen sinnvollen Ansatz darstellen kann, Resilienz in der Altenbevölkerung zu stärken. Diese könnte beispielsweise durch eine Verstetigung nachbarschaftlicher Unterstützungsangebote, wie sie zu Beginn der COVID-19-Pandemie vielfach initiiert wurden, gestärkt werden. Auch sollten insbesondere für Menschen in besonders hohem Alter Vernetzungsangebote wie Nachbarschaftscafés und Gemeinschaftsaktivitäten, ehrenamtliche Tätigkeiten oder auch gemeinschaftliche Wohnformen von kommunaler Seite gefördert werden.

Die hohe Bedeutsamkeit sozialer Kontakte deckt sich mit Befunden von Reissman et al. (2021) aus der NRW80+-Studie [49]. Sie konnten zeigen, dass das Wohlbefinden in der Altenbevölkerung ab 80 Jahren unter anderem bedeutsam mit interpersonaler Verbundenheit als Aspekt von Spiritualität zusammenhängt und diskutieren eine geringere Selbstbezogenheit im höheren Alter. In der Berliner Altersstudie wurde die Bedeutsamkeit funktioneller und gesundheitlicher Aspekte für das subjektive Wohlbefinden deutlich [20]. In künftigen Studien zu Korrelaten des Resilienzerlebens in der älteren Bevölkerung sollten neben sozialen Aspekten Spiritualität, Sinnempfinden und medizinische, funktionelle Faktoren einbezogen werden.

### Limitationen

Unsere Studienergebnisse stellen eine querschnittliche Analyse von Resilienzkorrelaten dar, deswegen können keine kausalen Schlüsse gezogen werden. Längsschnittstudien zur Resilienz bei älteren Menschen stehen aus [50]. Aufgrund der geringen Fallzahl der Menschen ab einem Alter von 85 Jahren in der LIFE-Adult-Studie konnten wir keine differenziertere Analyse von Alterseffekten innerhalb der Altenbevölkerung durchführen. Gerade in der Gruppe der Menschen ab dem Alter von 85 Jahren bestehen weiterhin hohe Forschungsbedarfe, welche in zukünftigen Studien adressiert werden sollten. Weiter ermöglichen die Ergebnisse keine Beurteilung der klinischen Bedeutsamkeit von Unterschieden in der Resilienz. Künftige Studien sollten untersuchen, inwiefern sich insbesondere bei älteren Menschen hohe bzw. niedrige Resilienz in der RS-11 widerspiegelt. Auch sollten zukünftige Studien (altersspezifische) Belastungsfaktoren, wie die häusliche Pflege von Angehörigen oder Multimorbidität, und mögliche Interaktionseffekte der soziodemografischen und sozialen Variablen miteinbeziehen.

## Fazit

Insgesamt legen unsere Ergebnisse nahe, dass die Resilienz von Menschen ab 65 Jahren mit jener der Allgemeinbevölkerung vergleichbar ist. Mit spezifischerem Blick auf soziodemografische Korrelate von Resilienz wird innerhalb dieser Altersgruppe eine geringere Resilienz bei über 75-Jährigen und bei Verheirateten deutlich. Diese Gruppen sollten in der öffentlichen Gesundheitsförderung verstärkt in den Fokus rücken, da insbesondere Letztere von Unterstützungsangeboten bisher wenig adressiert worden sind. Einen wichtigen Ansatzpunkt für die Resilienzförderung kann die Stärkung der sozialen Einbindung darstellen.
